# Dysentery, and Retroversion of the Uterus Considered as an Effect of the Same Cause

**Published:** 1853-10

**Authors:** R. R. Gresham

**Affiliations:** Ebenezer, Mississippi


Art. IV.—*Dysentevy, and Retroversion of the Uterus Considered as an Effect of the same cause.* By R. R. Gresham, M.D., of Ebenezer, Mississippi.

We had in 1852, in Latitude 32½° North, in Mississippi, dysentery, complicated and uncomplicated, which was referred to a peculiar malarious influence exercised over the systems of men, women, and children in this climate. But why it is that this epidemic spends its force upon the lower bowels we are unable to say, but that such is the fact is well known to every observing practitioner.

Whether the cause of this inflammatory action can remain latent in the system any length of time, and bring its influence to bear upon other organs through sympathy, is what we wish to inquire after now. The circumstances of a number of cases that came under my observation the past season, leads me to the conclusion that such may be the case. I have seen a number of cases of retroversio uteri occurring this season, which seemed connected with, or dependent upon, an irritation of pelvic viscera. And often have I witnessed cases of retroversion relieved apparently, followed by an inflammation of the lower portion of the alimentary canal, and an effort to throw off its contents.

The peculiar tendency of the uterine system to relieve itself of its burthen this season, has led to the foregoing remarks. And in reference to what I have already said, I believe, from observations made by myself, that we may have cases of retroversion dependent upon the same epidemic cause, not connected with, or followed by, inflammation of the bowels, for in 1852 we did not see any strong tendency of the uterus to relieve itself of its contents, while this year there is a strong tendency more or less on the part of the uterus to throw off its contents, which is owing to some unknown influence directed to the lower portion of the abdominal viscera. In eleven out of fourteen cases of observation, there was a tendency on the part of the uterus to abort, before any symptoms of dysentery were visible. All were cases of a retroversion, the result of a relaxed condition of the ligaments that support the uterus. After conferring with two of my seniors in the profession as to the nature of this disease, I have come to the conclusion that it is the effect of the same cause that produces dysentery or flux in this latitude, whether it occurs with or without irritation of the bowels.

But why was not a majority of similar cases last year attended with like results? or was it because the treatment was more palliative than it had been heretofore?

The only difference in my practice was a resort to cold applications externally, and enemas of cold water internally, in the place of blistering, and the various astringents.

It may be thought I am taking a half-way position between Allopathy and Hydropathy, as I on a former occasion eulogized the efficacy of cold water, but the phenomena and effect of the disease referred to, and the therapeutic application of cold water considered, has made it one of the most successful remedies in my hands.

If I succeed in calling the attention of some one to this subject, to make a further investigation, I shall have done all I designed in this communication.

SEPTEMBER 5, 1853.

